# Resveratrol, Metabolic Syndrome, and Gut Microbiota

**DOI:** 10.3390/nu10111651

**Published:** 2018-11-03

**Authors:** Alice Chaplin, Christian Carpéné, Josep Mercader

**Affiliations:** 1 Cardiovascular Research Institute, School of Medicine, Case Western Reserve University, Cleveland, OH 44106, USA; 2 INSERM U1048, Institute of Metabolic and Cardiovascular Diseases (I2MC) and University Paul Sabatier, 31432 Toulouse, France; christian.carpene@inserm.fr; 3 Department of Fundamental Biology and Health Sciences, University of the Balearic Islands, 07122 Palma, Spain; 4 Balearic Islands Health Research Institute (IdISBa), 07122 Palma, Spain

**Keywords:** resveratrol, gut microbiota, metabolic syndrome

## Abstract

Resveratrol is a polyphenol which has been shown to have beneficial effects on metabolic syndrome-related alterations in experimental animals, including glucose and lipid homeostasis improvement and a reduction in fat mass, blood pressure, low-grade inflammation, and oxidative stress. Clinical trials have been carried out to address its potential; however, results are still inconclusive. Even though resveratrol is partly metabolized by gut microbiota, the relevance of this “forgotten organ” had not been widely considered. However, in the past few years, data has emerged suggesting that the therapeutic potential of this compound may be due to its interaction with gut microbiota, reporting changes in bacterial composition associated with beneficial metabolic outcomes. Even though data is still scarce and for the most part observational, it is promising nevertheless, suggesting that resveratrol supplementation could be a useful tool for the treatment of metabolic syndrome and its associated conditions.

## 1. Introduction

In obese states, insulin resistance and inflammation are the underlying causes of the metabolic syndrome, and together with high blood triglycerides, altered cholesterol levels, glucose intolerance and hypertension, they greatly increase the risk of type 2 diabetes and cardiovascular disease [1]. Currently, the main treatments for metabolic syndrome are weight loss and physical activity; however, there is also evidence that pharmacotherapy (insulin sensitizers, statins, angiotensin-converting enzyme inhibitors) and, more recently, nutritional strategies could be beneficial [2]. Within this context, resveratrol, a natural phytochemical widely found in plants, fruits, and red wine, presents itself as a potential candidate, due to the recent observations in various animal models and human studies of its beneficial effects in terms of glucose and lipid homeostasis and reduced body fat accumulation [3,4,5,6]. It is known as a phytoalexin because of its ability to inhibit the progress of certain infections, and is one of the components of red wine believed to contribute to the French Paradox, which refers to a low prevalence of ischemic heart disease in populations with high intakes of saturated fat [7]. Furthermore, it has been demonstrated that resveratrol mimics calorie restriction effects through sirtuin 1 (SIRT1) activation, and thus extends the lifespan in simple organisms and prevents the deleterious effects of excess caloric intake in rodents, such as insulin resistance and body fat accumulation [4,8]. In humans, however, the effectiveness towards the treatment of metabolic syndrome has been reported to be in some cases lower than in experimental animals [9]. In addition, discrepancies and inconsistencies have been observed in clinical trials, which can be explained by differences regarding the form of supplementation and the characteristics of the treated individuals, such as age, sex, presence of single nucleotide polymorphisms, presence of metabolic disturbances, and gut microbiota composition. Considering resveratrol is metabolized by gut microbiota [10,11] and that it can influence its composition [12], the interplay between this stilbenoid and the host microbiota may strongly influence treatment efficiency, by either increasing its bioavailability, producing certain metabolites, or even by promoting the growth of specific bacteria. Thus, the resveratrol/microbiota interaction is a key element in the effectiveness of the treatment of metabolic syndrome. In this review, we aim to discuss the current knowledge regarding the effects of resveratrol on metabolic syndrome alterations and on gut microbiota and also try to determine how such interactions could modulate the beneficial effects of this phytochemical.

## 2. Resveratrol Occurrence, Absorption, Metabolism, and Bioavailability

Resveratrol (3,5,4’-trihydroxy-*trans*-stilbene) is a natural phytochemical widely found in its *trans* isomer form in various plants, such as *Polygonum cuspidatum*, in fruits, including grapes and berries, peanuts, and in red wine [13,14]. It is a polyphenolic compound of low molecular weight that belongs to the stilbenoid family of polyphenolic compounds (hydroxylated derivatives of stilbene based on a C_6_-C_2_-C_6_ polyphenolic structure). Resveratrol naturally occurs in these sources mainly in its glycosylated form, known as piceid and polydatin (3,4’,5-trihydroxy-stilbene-3-β-mono-d-glucoside). Its use as a nutraceutical has been studied in both animal and human models, including clinical trials, in the context of obesity, metabolic syndrome, heart disease, and cancer, among others; however, to date, there are no specific recommendations concerning dosage and length of supplementation. 

Upon ingestion, resveratrol or its precursors travel through the gastrointestinal tract, and it is estimated that around 70% of the intake of resveratrol is absorbed [13]. In the intestine, resveratrol binds to several nutrients, such as proteins, and the solubility of these will influence its absorption or elimination in feces [13]. Resveratrol absorption occurs by passive diffusion or by forming complexes with intestinal membrane transporters, including integrins. Free resveratrol circulates in the bloodstream bound to lipoproteins and albumin. However, the free form of resveratrol is found at very low levels in the bloodstream due to extensive glucuronidation in the liver and intestine and sulfation in the liver, thereby decreasing its bioavailability [14]. Hence, the major circulating forms of resveratrol are glucuronide (*trans*-resveratrol-3-glucoronide, *trans*-resveratrol-4’-glucuronide) and sulfate (*trans*-resveratrol-3-sulfate, *trans*-resveratrol-3,4’-disulfate, *trans*-resveratrol-3,5-disulfate) conjugate metabolites [13]. Likewise, resveratrol-3-sulfate and resveratrol-3-glucuronide are detected in target organs, such as in the liver, adipose tissue or the heart, after oral administration [15,16].

Besides resveratrol conjugates, other resveratrol derivatives are also detected in target tissues, such as piceatannol and dihydroresveratrol [17,18]. The occurrence of piceatannol (3,3’,4,5’-tetrahydroxy-*trans*-stilbene) results from resveratrol hydroxylation in the liver by cytochrome P450 [18] and stands out due to its potentially beneficial effect on the metabolic syndrome [19]. It is a more stable stilbenoid that also naturally occurs in diverse plants. Dihydroresveratrol, synthesized by gut bacteria, together with free resveratrol, are detected in tissues after sustained resveratrol administration, whereas glucuronide and sulfate are the main resveratrol metabolites detected in tissues after an acute administration of resveratrol [17]. Therefore, the bioavailability of resveratrol and its metabolites largely differs on whether the administration is acute or sustained. In addition, the bioavailability of resveratrol and its metabolites is dose-dependent [15,16].

In turn, free resveratrol can be synthesized back from its sulfate and glucuronide derivatives by ubiquitously expressed sulfatase and β-glucuronidase [13]. Moreover, resveratrol metabolites can return to the small intestine through the bile enterohepatic transport. So far, up to 21 resveratrol metabolites have been identified in human urine after moderate consumption of red wine for 28 days [20], and it has been observed that some of these metabolites are by-products of gut microbial metabolism.

### Impact of Gut Microbiota on Resveratrol Metabolism

**Figure 1:**
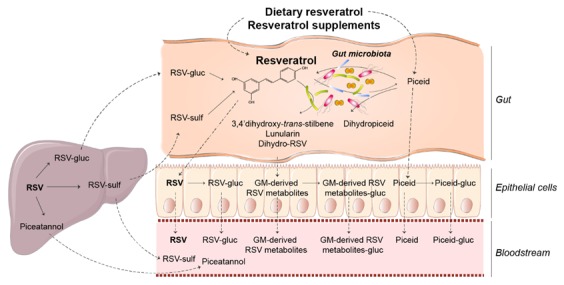
Overview of the metabolism of resveratrol and the impact of gut microbiota. Upon intake, RSV and RSV precursors enter the gut and are partly metabolised by gut microbiota to produce particular RSV derivatives and RSV. Free RSV, RSV precursors and microbiota-derived RSV metabolites are conjugated in the intestine and liver, from where conjugated forms can return to the intestine. In the liver, RSV is metabolised to piceatannol, which can be released into the bloodstream, and delivered to target tissues together with RSV, RSV precursors, microbiota-derived RSV metabolites and their respective conjugate forms. RSV, resveratrol; gluc, glucuronides; sulf, sulfates.

The fact that the gut microbiota is highly responsible for metabolizing resveratrol has been known for some time; however, the importance of this process and of the metabolites and other by-products derived from this is only starting to gain relevance. As illustrated in Figure 1, the gut microbiota actively participates in resveratrol metabolism by increasing its availability from resveratrol precursors and producing resveratrol derivatives.

Gut bacteria metabolize resveratrol precursors to resveratrol, thereby increasing its bioavailability [14,21]. In particular, gut bacteria hydrolyze the glucoside moieties of plant glycosydes, such as the resveratrol precursor piceid, and thus externalize their aglycones. *Bifidobacteria infantis* and *Lactobacillus acidophilus* are two bacteria responsible for resveratrol production from piceid [11,21,22]. In turn, resveratrol can be glycosylated in the gut to produce piceid again. Piceid is conjugated to piceid glucuronide, and can be absorbed in both its free form, and most abundantly, in its conjugated form [23].

On the other hand, gut bacteria metabolize resveratrol and its precursors, resulting in certain resveratrol derivatives. Dihydroresveratrol was the first derivative identified, which is produced by *Slackia equolifaciens* and *Adlercreutzia equolifaciens*, followed by two other bacterial *trans*-resveratrol metabolites, 3,4’-dihydroxy-*trans*-stilbene and 3,4’-dihydroxybibenzyl (lunularin) [10]. Gut bacteria also metabolize piceid to produce dihydropiceid and dihydroresveratrol [22]. As resveratrol, gut bacteria-derived resveratrol derivatives are also conjugated to its glucuronide forms.

Furthermore, higher concentrations of dihydroresveratrol glucuronides than resveratrol glucuronides and glucosides have been found in human plasma and urine after the intake of a grape extract or red wine [23]. In tissues of rats receiving resveratrol, dihydroresveratrol glucuronide is also detected in the liver, whereas dihydroresveratrol sulfate is detected in the liver and adipose tissue [16]. The amount of dihydroresveratrol sulfate in the liver is remarkably higher than that of resveratrol sulfate. These observations highlight the importance of gut microbiota in resveratrol metabolism, particularly in producing specific resveratrol derivatives in significant amounts.

## 3. Effect of Resveratrol on Metabolic Syndrome

Metabolic syndrome is a cluster of a least three of the five following metabolic alterations: Central obesity, high blood glucose, high blood pressure, high serum triglycerides, and low serum high-density lipoprotein (HDL), the presence of which increases the risk of developing cardiovascular disease and type 2 diabetes [1]. Here, we describe the potential effect of resveratrol supplementation on each metabolic alteration and review the clinical trials that analyze the effects on them. It is important to note that there are still many discrepant and inconsistent results in human studies and it is clear that further research needs to be carried out to further understand the effect of resveratrol on metabolic syndrome.

### 3.1. Fat Accumulation

Over a decade ago, it was discovered that resveratrol can reduce diet-induced obesity through SIRT1 activation, generating high expectations as a potential anti-obesity molecule; since then, this effect has been shown in both mice and rats [4,8,24,25,26]. SIRT1 activation is of interest because it deacetylates and activates peroxisome proliferator-activated receptor (PPAR) γ coactivator 1α [4], which controls mitochondrial biogenesis and function. Furthermore, it triggers lipolysis and loss of fat by repressing PPARγ in adipocytes [27]. Resveratrol also acts by inhibiting cAMP-specific phosphodiesterases, leading to elevated cyclic adenosine monophosphate levels, which in turn activate adenosine monophosphate-activated protein kinase (AMPK) [28]. Other anti-lipogenic mechanisms of action have been described, including the upregulation of certain microRNAs by resveratrol, which leads to the inhibition of lipogenesis in white adipose tissue [29].

#### 3.1.1. Resveratrol and Adipose Depot Extension

Since the discovery of its actions on SIRT1, many other studies have been carried out to determine the potential of resveratrol in the treatment of obesity. Studies carried out in cell systems so far have shown that the anti-obesity effect of resveratrol is attributed to its actions on (1) pre-adipocytes, including the induction of apoptosis and the inhibition of proliferation and differentiation [30,31]; (2) mature white adipocytes, including the activation of lipolysis, inhibition of *de novo* lipogenesis, and promotion of brown adipocyte features [30,32,33,34,35]; and (3) brown adipocytes, in which the expression of the uncoupling protein-1 and typical brown-phenotype genes are induced [36]. Studies in rodents show that the resveratrol-induced reduction in fat mass is partly explained by both the activation of lipolysis through the adipose triglyceride lipase, and the inhibition of *de novo* lipogenesis by controlling the expression of the lipogenic enzymes mediated by the transcription factor, sterol regulatory element-binding protein-1c [25,29,37,38]. Moreover, the activation of brown adipose tissue and the induction of brown-like adipocytes in white adipose tissue (browning) seem to contribute to body fat reduction in resveratrol-treated rodents [34,36,39].

In humans, however, most of the research published until now shows a lack of effect on body adiposity and body weight. *Trans*-resveratrol supplementation at doses between 100 and 500 mg for a 4-week to 6-month period did not affect fat mass or body weight in obese healthy individuals [32,40,41]. Despite this, adipocyte size was reduced, meaning that adipose tissue was modified by resveratrol intake, even if to a small degree [42]. Furthermore, two studies looking into the effect of resveratrol supplementation in patients with metabolic syndrome showed discrepant results; while Kjœr et al. [43] reported no effects on body composition with doses of 150 mg and 1000 mg, Méndez del Villar et al. [44] showed a reduction in fat mass, waist circumference, body mass index, and body weight after receiving 500 mg three times a day. However, patients with non-alcoholic fatty liver disease (NAFLD) or type 2 diabetes showed no changes regarding fat mass or body weight after resveratrol intake [45,46,47,48]. Moreover, other studies looking into the effect of resveratrol combined with other molecules, such as the intestinal lipase inhibitor orlistat [32] or epigallocatechin-3-gallate (EGCC) [49], did not show significant changes in fat mass.

#### 3.1.2. Resveratrol and Hepatic Fat Accumulation

A lifestyle pattern including unbalanced diets and sedentary behavior promotes the accumulation of ectopic fat, particularly in the liver. As in metabolic syndrome, visceral adiposity and the pro-inflammatory state are also key in the development of NAFLD. In view of the inhibitory effects of resveratrol on fat accumulation, many studies have addressed its effect on hepatic fat and the management of NAFLD. Interestingly, hepatic triacylglycerol and cholesterol content is reduced by resveratrol supplementation in rodents fed a high-fat diet [26,50,51,52,53,54,55]. An increased number of mitochondria and fatty acid oxidation activity and a decreased lipogenic activity seem to be involved in normalizing hepatic steatosis in rodents [50,51,55], in addition to the anti-oxidant and anti-inflammatory action of the polyphenol [53,54]. In humans, resveratrol supplementation was also shown to reduce intrahepatic lipid content in one study involving obese individuals [41], but not in another [40]. Furthermore, when considering patients with NAFLD, clinical trials are still insufficient to demonstrate a clear positive effect of resveratrol according to the conclusions of two meta-analyses, which show a lack of effect on NAFLD features [56,57]. Likewise, hepatic lipid deposition is unaffected in individuals with type 2 diabetes [58] or metabolic syndrome [43].

### 3.2. Resveratrol and Glucose Intolerance and Insulin Resistance

As it was demonstrated that resveratrol improves insulin sensitivity by activating AMPK [4], many later studies have evaluated the use of the polyphenol in the management of glucose control and in type 2 diabetes mellitus, the risk of which is increased under the persistence of glucose intolerance. Studies in cultured cells and animals have further contributed to the understanding of the mechanism of action of resveratrol on glycemic control and insulin resistance. Resveratrol activates the insulin-signaling components insulin receptor substrate-1 and Akt [59,60] and reduces the expression of adipokines that influence insulin sensitivity, including adiponectin [61], resistin, and retinol-binding protein 4 [33]. Resveratrol enhances insulin-stimulated glucose uptake in cultured cells [30] and, in vivo, reduces glycemia, insulinemia, and improves insulin resistance in diet-induced insulin-resistant mice [4,60,62]. Furthermore, the polyphenol protects from diabetic complications, such as diabetic nephropathy, diabetic retinopathy, and diabetes-induced hypertension [63,64,65,66].

When looking at the effects of resveratrol in a human setting, clinical trials have been conducted to elucidate its potential action on glucose homeostasis in individuals with different degrees of alteration in glucose homeostasis, from normoglycemia to type 2 diabetes. In non-diabetic individuals, four studies reported no changes in insulin sensitivity and insulin and glucose levels after resveratrol supplementation at doses between 75 and 2000 mg [40,67,68,69]. However, in other studies, a decrease in circulating glucose, an improved homeostatic model assessment–insulin resistance (HOMA-IR) score and a suppression in postprandial glucagon response were observed after supplementation with a 150-mg resveratrol dose for 30 days in obese non-diabetic individuals [41,70]. However, in type 2 diabetic patients, it was ineffective in improving insulin sensitivity following the same supplementation protocol. It was speculated that the lack of effect could be due to the interaction found between metformin and dihydroresveratrol levels [58]. The intake of higher doses of resveratrol did not affect the circulating levels of glucose, insulin, glycosylated hemoglobin, and glucagon-like peptide-1 in type 2 diabetic patients [47,48], as well as glucose tolerance and insulin sensitivity in older glucose-intolerant adults [71]. A beneficial effect in glucose intolerant or type 2 diabetic patients is, on the other hand, described in other studies [72,73,74,75], even at low doses (10 mg) [73]. Furthermore, in individuals with NAFLD, no changes in insulin resistance were observed in several studies [45,46,57], whereas glucose levels and insulin resistance were improved in one study [76]. Likewise, mixed outcomes are also reported in individuals with metabolic syndrome; while Méndez del Villar et al. [44] reported a decreased insulin response to glucose and total insulin secretion without affecting glucose levels, Kjœr et al. [43] reported no beneficial effects and actually found an increase in circulating fructosamine levels. Several meta-analyses have been carried out to shed light on the inconsistency of the above-mentioned results. In an early meta-analysis of 11 studies, Liu et al. concluded that there was no effect on glycemic measures in nondiabetic participants [77]. Among type 2 diabetic patients, Hausenblas et al. [78] identified a beneficial effect on hemoglobin A1c, but not on glucose, insulin, and HOMA-IR. More recently, a meta-analysis that included nine studies showed a beneficial effect on glucose and insulin levels and HOMA-IR, which was particularly more favorable for doses ≥100 mg/day [6]. Despite the discrepant results, the sample size, and the duration of the trials, it concluded that resveratrol might be used for treating diabetes, alone or in combination with current anti-diabetic therapies [66,79].

### 3.3. Resveratrol and High Blood Pressure

Excessive weight is linked to high blood pressure, which is a major risk factor for cardiovascular disease. Resveratrol supplementation reduces blood pressure in animal models of hypertension, including plexiglas clip- [80], angiotensin II- [81], or hypoxia-induced hypertensive rats, and in fructose-fed rats [82]. Several mechanisms are involved in the modulation of blood pressure by resveratrol, including AMPK phosphorylation, increased nitric oxide (NO) levels, SIRT1 activation, and decreased reactive oxygen species (ROS) production by regulating nicotinamide adenine dinucleotide phosphate oxidase, superoxide dismutase 2, and glutathione reductase [80,81,82,83]. In clinical trials, a reduction in blood pressure by resveratrol supplementation has been reported in individuals with obesity [41], NAFLD [84], or type 2 diabetes [72,85]; however, such an effect has not been found in other studies involving subjects with obesity [40,86], NAFLD [46], or metabolic syndrome [43]. Furthermore, several meta-analyses of randomized controlled trials show no significant effect of resveratrol supplementation on systolic and diastolic blood pressure [5,87], even though subgroup and meta-regression analyses indicate that resveratrol intake reduces systolic blood pressure and diastolic blood pressure at doses higher than 150–300 mg/day [5,88,89]. According to these analyses, a beneficial effect of resveratrol supplementation on blood pressure is observed when the effect is analyzed among diabetic patients [89] or overweight and obese individuals [88]. Moreover, despite reporting no changes in blood pressure, resveratrol supplementation improves endothelial dysfunction in obese subjects [86], which is also seen in hypertensive patients [90] and individuals with mild hypertension [91].

### 3.4. Hypertriglyceridemia

Excessive fat intake and the persistence of increased adiposity can lead to dyslipidemia, which increases cardiovascular risk. Studies have been carried out evaluating the effect of resveratrol on circulating lipids, particularly triacylglycerides and cholesterol, offering interesting results. Resveratrol supplementation reduces triglyceridemia in diet-induced obese rodents [25,26,54,55], which can be partly explained by the inhibition of hepatocyte fatty acid and triacylglycerol synthesis described in rat hepatocytes [92]. In humans, a reduction in triglyceridemia is observed when resveratrol is provided within a grape extract [93], mixed in a nutraceutical formula [94], or combined with other molecules, such as epigallocatechin-3-gallate (EGCC) [49] or orlistat [32]. When resveratrol is provided alone, it reduces plasma triglyceride levels in individuals with dyslipidemia [95] or obesity [58]. However, in other studies carried out in individuals with obesity [67], type 2 diabetes [74], NAFLD [46] or hypertriglyceridemia [68], resveratrol does not influence triglyceridemia, which is confirmed by Sahebkar’s meta-analysis [87]. Furthermore, other studies actually report an increase in triglyceridemia, including Haghighatdosst’s meta-analysis of 20 studies [3,48].

### 3.5. Altered Cholesterolemia

The effect of resveratrol on circulating levels of total, low-density lipoprotein (LDL), and HDL cholesterol has been evaluated. It is thought resveratrol may affect cholesterolemia by increasing the synthesis and efflux of bile acids, decreasing the synthesis of hepatic cholesterol, and increasing the efflux of cholesterol [96,97,98]. In this context, it has been shown that resveratrol supplementation reduces total cholesterol levels in diet-induced obese rodents [25,26,54,55,97], whereas mixed results are reported in humans, showing either a lack of effect [67] toward the reduction [72,88,95], and even an increase in total cholesterol levels [43,48], as described for triglyceridemia. A lowering effect of resveratrol supplementation on plasma LDL and total cholesterol concentrations has been reported in studies in which the compound is given within a plant extract or when combined with a nutraceutical formula [93,94,99], in which it was concluded that the presence of resveratrol is necessary to achieve this effect [99]. A recent meta-analysis of randomized clinical trials that used resveratrol as a mono food supplement concluded that the compound had no effect on the circulating levels of total, LDL, or HDL cholesterol [3]. In addition to its potential ability to influence cholesterol levels, resveratrol has been shown to inhibit LDL and HDL oxidation in vitro [98] and to reduce plasma oxidized LDL cholesterol levels [99].

### 3.6. Inflammation and Oxidative Stress

#### 3.6.1. Inflammation

Studies have shown that resveratrol exerts an anti-inflammatory activity, and have demonstrated its capacity to inhibit the production of pro-inflammatory cytokines, as well as the activity of cyclooxygenases (COX)-1 and -2 and inducible NO synthase. This anti-inflammatory effect is mainly mediated by the ability to inhibit the transcriptional activity of nuclear factor kappa beta (NF-κβ) and activator protein-1 [100], and can also be attributed to the modulatory effect of microRNAs expression with either an anti-inflammatory or a pro-inflammatory role [101]. Within this context, it has been revealed that resveratrol can decrease chronic low-grade inflammation, which is characterized by adipose tissue macrophage accumulation and abnormal cytokine production. For example, in murine adipocytes and human adipose tissue explants it decreases the secretion of monocyte chemoattractant protein-1 [102], tumor necrosis factor-α (TNF-α), and interleukins (Il)-1β, -6, and -8 [30,103,104], as well as the production of prostaglandin E2 [105], and the expression of vascular endothelial growth factor [106]. These effects are observed in cells treated with TNF-α or Il-1β, exposed to hypoxic conditions, or treated with the microbial product lipopolysaccharide (LPS) [59,107]. In vivo, resveratrol intake reverses obesity-associated inflammation in genetically-induced obese rats [108] and diet-induced obese rodents and monkeys [25,54,55,109,110,111,112]. Furthermore, several human studies have reported that resveratrol can even have an acute anti-inflammatory effect in healthy subjects. For example, the intake of a single grape extract reduces plasma IL-1β levels induced by a high-fat and high-carbohydrate meal and, interestingly, plasma endotoxin levels [113]. In type 2 diabetic and hypertensive patients, long-term grape extract supplementation reduces serum Il-6 and alkaline phosphatase levels and alters the expression of pro-inflammatory genes and microRNAs in peripheral blood mononuclear cells [114]. However, in a study involving type 2 diabetic patients, 800 mg/day resveratrol supplementation did not change plasma levels of inflammatory cytokines [115]. Meta-analyses of randomized controlled trials indicate that resveratrol treatment reduces the levels of C-reactive protein and that of TNF-α among obese subjects, confirming the anti-inflammatory action of resveratrol [116]. Interestingly, the pro-inflammatory status which occurs in diabetic complications, such as diabetic neuropathy and nephropathy, seems to also be inhibited by resveratrol [64,117].

#### 3.6.2. Oxidative Stress

Resveratrol exerts an anti-oxidant action, which underlies the beneficial effect of this polyphenol on several metabolic disturbances, such as glucose intolerance, insulin resistance, and hepatic fat accumulation. The mechanisms of action of its anti-oxidant effects include direct mechanisms, such as neutralizing ROS and reactive nitrogen species, and indirect mechanisms such as the ability to increase the transcriptional activity of nuclear factor-E(2)-related factor-2 (Nrf2) and forkhead box O [118]. Experiments on cell cultures designed to study the effect of resveratrol on metabolic syndrome alterations, particularly by exposing cells to a high glucose concentration or to pro-inflammatory cytokines, show a reduction in ROS levels in many cell types, including in vascular endothelial cells [119,120], adipocytes [104], monocytes [121], and cardiomyocytes [122]. In obese and/or diabetic rodents, a reduction in oxidative stress accompanies the improvement of inflammation [55,110,112], hyperglycemia and insulin resistance [62,112], diabetic nephropathy [63], fat mass accumulation [112], hepatic steatosis [52,53,55,123], hypertriglyceridemia [55], hypercholesterolemia [55], endothelial function [124,125], ventricular diastolic relaxation [126], and hypertension [123]. In humans, the intake of resveratrol reduces oxidative stress in both healthy individuals and patients with metabolic diseases that are characterized by a high oxidative stress degree. The intake of a resveratrol supplement or resveratrol-containing extract increases the total antioxidant capacity and reduces oxidative stress in healthy individuals [93,94,127], as well as oxidative stress generated by the intake of a meal rich in fat [113]. In type 2 diabetic patients, resveratrol supplementation reduces markers of oxidative stress, which are accompanied by an improvement of insulin sensitivity, blood pressure, and cardiovascular function [73,85]. The improvement in insulin sensitivity and diabetic complications caused by resveratrol is explained by its ability to reduce oxidative stress [64,65,73]. Since resveratrol is a relatively unstable molecule, strategies aimed at increasing its stability and thus enhancing its inhibitory action on oxidative stress have been developed. In this context, an enhanced reduction in oxidative stress in obese individuals has been achieved by the intake of a more stable resveratrol derivative [128].

## 4. Role of Resveratrol Metabolites in Metabolic Syndrome

Most of the studies dealing with the potential beneficial effects of resveratrol on metabolic syndrome use *trans*-resveratrol. However, in vivo effects cannot be solely attributed to this molecule, as it is likely that resveratrol metabolites are also involved. As detailed above, upon intake, several resveratrol metabolites can be produced in the body, including piceid, glucuronide, and sulfate resveratrol conjugates, dihydroresveratrol and other derivatives produced by gut microbiota, and piceatannol, among others. One of the most studied resveratrol metabolites is piceid, which shows a higher bioavailability than resveratrol. As described for resveratrol, piceid shows anti-oxidant and anti-inflammatory activities and shares with resveratrol many of the described molecular targets, including SIRT1, NF-κβ, and NRF2. Interestingly, its anti-oxidant activity is higher than that of resveratrol [129]. In vivo, piceid treatment reduces insulin resistance, steatosis, and dyslipidemia [130,131,132]. Regarding resveratrol conjugates, there are little data on their potential metabolic effects. Resveratrol glucuronides and sulfates inhibit triacylglycerol accumulation in differentiating adipocytes and adipokine expression in mature adipocytes [133,134]. Additionally, resveratrol glucuronides seem to have a greater potential to lowering the effect of cholesterol [96] compared to resveratrol, whereas resveratrol sulfates inhibit NO production and exert a differentiated effect when compared to glucuronides on free radical scavenging and COX activity, NF-κβ induction, and pro-inflammatory cytokine expression inhibition [135,136]. It would be interesting to elucidate whether the resveratrol metabolites produced by gut bacteria are able to trigger beneficial effects, particularly bearing in mind the high concentrations of dihydroresveratrol and its derivatives detected in plasma and tissues [16,23]. Only limited data exist regarding the effects of dihydroresveratrol, 3,4’-dihydroxy-*trans*-stilbene, and lunularin in parameters related to metabolic syndrome. Dihydroresveratrol exhibits significant antioxidant activity [137], reduces fatty acid-binding protein-4 expression, involved in fatty acid uptake in human macrophages treated with oxidized LDL [138] and stimulates fatty acid oxidation in human fibroblasts [139]. Lunularin reduces the expression of pro-inflammatory mediators in endothelial cells in response to LPS [140], and 3,4’-dihydroxy-*trans*-stilbene activates AMPK, induces glucose uptake in C2C12 myotubes, and reduces PPARg and resistin expression in 3T3-L1 adipocytes, showing a larger effect than resveratrol [141]. Collectively, these results suggest that gut bacteria-derived resveratrol metabolites could be involved in the effect of resveratrol supplementation on metabolic syndrome alterations, and consequently, gut bacteria amount and composition may be determinant. A resveratrol-related stilbenoid with reported effects against metabolic syndrome alterations is piceatannol, which inhibits fatty acid-induced inflammation and oxidative stress and reduces hyperlipidemia, hyperglycemia, and insulin resistance [19,100,142,143,144]. Research has thus focused on this stilbenoid due to its higher stability and absorption compared to resveratrol [145], which is associated with a higher anti-inflammatory activity versus resveratrol [146]. Moreover, differential effects between these stilbene derivatives have been shown regarding their anti-lipolytic activity, being stronger for piceatannol, which was linked to its inhibitory action on lipotoxicity [143]. Overall, it seems that resveratrol metabolites could be involved in some of the beneficial effects attributed to resveratrol with regards to the metabolic syndrome alterations. The fact that the gut microbiota plays an important role in the conversion and/or production of some of these resveratrol-related metabolites has led to the idea that it could actually be modulating such described effects.

## 5. The Role of Gut Microbiota in Health and Disease

The gut microbiota, including its role in health and disease, is currently one of the topics of highest interest in biomedical research, due to its potential key role in the aetiology and development of many diseases [147,148]. In the last decade, it has been associated with conditions such as obesity, diabetes, cardiovascular disease, and cancer, which are among the leading causes of mortality and morbidity worldwide [149,150,151,152,153]. More recently, the hypothesis that the gut microbiota is one of the key modulators that influence disease risk due to its close links to metabolism and the immune system has been posed [154]. It was even coined as “the forgotten organ” initially [155], due to the vast amount of processes it is involved in, including the processing of non-digestible polysaccharides from the diet into short-chain fatty acids (SCFA) [156], synthesis of vitamins, and regulation of energy balance and immune functions [147,157]. The term “gut microbiota” refers to the bacteria, archaea, and eukarya found in the gastrointestinal tract [158]. It is widely thought that the number of microorganisms greatly outnumber human cells (with a suggested ratio of 1:10) [159], and 100 times the amount of genomic content (“gut microbiome”) [158], which inevitably leads to the assumption that they carry out a major role in the body; interestingly, this calculation was recently challenged and the idea that the ratio may be closer to 1:1 was put forward [160], without underestimating their impact on human health. This new focus on the role of gut microbiota on health and disease has led to large studies worldwide, including the Human Microbiome Project [161], which was essentially an extension of the Human Genome Project, and the MyNewGut project, which is currently ongoing and focuses on the role of the microbiome in the development of diet and brain-related disorders, among others. The ultimate goal of this new “gut microbiota era” is to understand what bacterial composition defines a healthy gut, and how this knowledge can be translated into efficient and targeted therapies for diseases in which it seems to be playing a main role.

Initially, it was thought that humans could be divided into three main enterotypes based on the make-up of their gut microbiota composition, focusing on one of three dominating genera: Bacteroides, Prevotella, and Ruminococcus [162]; since then it has been shown that gut bacteria are easily modified by many factors, including delivery method, diet, lifestyle, medication use, and infections [148,163,164,165], making an individual´s enterotype variable throughout their lifespan and hence the use of bacterial clusters as biomarkers for disease not as effective as previously thought [166]. Although it is still being debated what constitutes a “healthy” gut microbiota composition, it has been widely established that dysbiosis, which refers to a disturbance in the amount and/or composition of an individual’s “normal” gut microbiota, is strongly associated to many common diseases [150,152,154,163]. People presenting certain conditions, such as obesity and metabolic syndrome particularly, consistently present a lower bacterial diversity and composition compared to their healthy counterparts [153,163,167,168,169,170,171]. Furthermore, it was recently hypothesized that since a more diverse microbiota translates into carrying more genes and is involved in more metabolic pathways, it is better prepared to adapt to changes in diet and thus the host could respond better to dietary treatment [172].

### 5.1. The Impact of Gut Microbiota on Energy Metabolism

As previously discussed, the potential modulation of energy homeostasis by the gut microbiota has been of great interest, with studies indicating significant differences between the gut microbiota of obese versus lean subjects. Many animal studies are offering potential mechanistic views on how the gut microbiota operates, and although this proves to be more challenging in a human setting [173], evidence continues to point to the “forgotten organ” as one of the key players.

Over a decade ago, the first data emerged showing that germ-free mice weighed significantly less and had a lower amount of body fat [174,175,176]. Since then, numerous studies have shown that the gut microbiota composition in obesity is different compared to lean subjects, characterized by increased levels of Firmicutes and less Bacteroidetes [177,178], increased capacity in energy harvesting from dietary polysaccharides [175,179], and is associated with increased adiposity and insulin resistance [180,181] and lower levels of short-chain fatty acids in the caecum [173].

Furthermore, more and more evidence points towards the significant role of systemic and adipose tissue inflammation in the development of obesity, diabetes, and metabolic syndrome, thus many studies soon began to investigate whether gut microbiota could be contributing to it [182,183,184,185]. It has been shown that LPS, found on the outer membrane of Gram-negative bacteria, triggers inflammatory pathways by binding the CD14/Toll-like receptor-4 complex and that chronic high levels in plasma lead to insulin resistance and diabetes [185,186,187]. Hence, it is hypothesized that certain factors (such as diet) can promote Gram-negative bacteria in the gut, promoting leakage of LPS through the gut epithelium and thus leading to an increase in plasma LPS levels, inducing insulin resistance and what is known as metabolic endotoxaemia [172,188].

Thus, it seems that the gut microbiota has a big impact at both the peripheral and the central level with regard to overall energy regulation, and hence it is being considered as a potential therapeutic strategy for subjects who present obesity, diabetes and/or metabolic syndrome. One of the key ways to manipulate gut microbiota is through the diet, as discussed in the next section, with a focus on the influence of natural polyphenols.

### 5.2. Diet as a Key Modulator of the Gut Microbiota

Data so far point to the gut microbiota and its metabolic products as a key player in obesity and metabolic syndrome [149,150,152], hence the next step at present is to determine plausible ways in which to manipulate bacterial composition in order to impact host physiology in a beneficial manner. Evidence suggests that gut microbiota may be the link between diet and obesity development, due to its capacity for changing microbial composition and activity in the gut, as seen in both mice and humans [149,150,152]. Therefore, it is essential to consider diet as an important factor when designing new therapies and prevention strategies regarding obesity [189,190,191].

Dietary habits on the whole have a significant role in shaping the gut microbiota–this is evidenced by the fact that individuals of different countries have distinct bacterial populations [192]. One of the first studies to publish this was carried out by De Filippo et al., which showed that children in a rural African village had low levels of Firmicutes and high levels of Bacteroidetes in fecal samples compared to Italian children, who presented high levels of Enterobacteriaceae [193].

Furthermore, even though gut microbiota is relatively stable throughout adulthood in humans [194], studies have shown that it can be rapidly modified by diet [195], with composition changes seen in as little as a few days of dietary intervention [174]. However, it seems this can be rapidly reversed, hence it has been suggested that a long-term (dietary) intervention may be needed in order to observe a significant shift in the enterotype of an individual [196].

Research has mainly focused on the effect of certain dietary patterns, such as high-fat and/or “Western” diets, which lead to decreased bacterial diversity, high numbers of Firmicutes and Proteobacteria, and low Bifidobacteria levels, which in turn are associated to a wide array of conditions, particularly obesity [196,197,198]. Other dietary interventions, such as the use of fructans, high-fiber, specific nutrients or prebiotics have been shown to promote bacterial diversity and increase Bifidobacteria in the gut, and thus having an overall beneficial effect on the host, such as decreasing low-grade inflammation [167,195,198,199].

Within the study of specific macro and micronutrients, we find an increased interest in the effects of molecules such as polyphenols, which have the ability to cause a significant shift in gut microbiota [12]. Here we will focus particularly on the effect of resveratrol has on the gut microbiota and how this knowledge could be used in the context of obesity and the metabolic syndrome.

### 5.3. Resveratrol and Gut Microbiota

Owing to the low bioavailability of resveratrol, it has been postulated that one of its mechanisms of action is through its interaction with the gut [200]. Recent studies have shown that resveratrol induces changes in the gut microbiota, which could lead to lower body weight and body fat, together with improved glucose homeostasis and obesity-related parameters. It seems this could be either by directly modulating bacterial populations to promote a composition associated with a healthy phenotype, or by the action of its by-products, which could be having an impact on genes and pathways involved in energy regulation. A summary of the studies discussed in this section can be found in Table 1.

#### 5.3.1. Effects of Resveratrol on Body Weight and Fat Metabolism

As previously discussed in this review, resveratrol could have a beneficial effect on body weight and body fat regulation based on evidence obtained in experimental animals, and studies are starting to point towards a potential implication of the gut microbiota as reviewed in Table 1. One study showed that C57Bl/6J mice on a high-fat diet and receiving 200 mg/kg/day of resveratrol supplementation through oral gavage five times a week (for a total of eight weeks) presented reduced fat deposition and body weight gain compared to controls receiving a high-fat diet alone [205]. In order to understand the potential mechanism through which resveratrol acts, the authors show that resveratrol activated the mammalian target of the rapamycin (mTOR) complex 2 (mTORC2) signalling pathway and inhibited mTORC1, a key player in energy regulation, suggesting that this suppresses the presence of obesity-associated gut microbiota, including *Lactococcus*, *Clostridium XI*, *Oscillibacter*, and *Hydrogenoanaerobacterium*.

In tune with these results, Kunming mice on a high-fat diet supplemented with the same amount of resveratrol (200 mg/kg/day) for 12 weeks also showed decreased body fat and weight by the end of the experiment [207]. These parameters were correlated with changes in gut microbiota, since resveratrol significantly increased *Lactobacillus* and *Bifidobacterium* (negatively correlated with body weight), and decreased *Enterococcus faecalis* (positively correlated with body weight). Furthermore, they showed a higher abundance of Bacteroidetes and a lower amount of Firmicutes bacteria (a ratio which was negatively correlated with body weight). Although the authors suggest that resveratrol could be having a prebiotic effect on bacteria, and that this could be having a positive effect on body weight and fat mass, further studies are needed to identify the potential mechanisms through which resveratrol acts.

Other studies have looked at the effect of resveratrol supplementation together with other compounds of interest. For example, one group administered a combination of quercetin (30 mg/kg body weight) and resveratrol (15 mg/kg body weight) by oral gavage per day to Wistar rats on a high-fat diet for 10 weeks [209]. By the end of the experiment, animals receiving supplementation presented lower body weight gain and adipose tissue weight compared to animals on a high-fat diet alone. Interestingly, they also had decreased Firmicutes and a lower Firmicutes to Bacteroidetes ratio, as well as increased levels of *Bacteroidales S24-7 group*, *Christensenellaceae*, *Akkermansia muciniphila*, *Ruminococcaceae, Ruminococcaceae UCG-014*, and *Ruminococcaceae UCG-005*, which have all been associated with reducing high-fat-diet-induced obesity.

In line with these results, it seems resveratrol could be a promising compound for use in promoting a healthy gut microbiota, which is known to have a wide array of beneficial effects. However, to the best of our knowledge, very few studies have been carried out which investigate the effects of resveratrol supplementation on gut microbiota in humans and the results obtained were slightly milder than those observed in animals. A recent study gave both males and females a combination of epigallocatechin-3-gallate (282 mg/day) and resveratrol (80 mg/day) supplements for 12 weeks; by the end of the experiment, they observed only slight differences in the gut microbiota composition of men only, with a minor reduction in Bacteroidetes and *Faecalibacterium prausnitzii* [213]. However, an increase in fat oxidation and skeletal muscle mitochondrial oxidative capacity was observed associated with supplementation and, interestingly, the Bacteroidetes level was correlated with fat oxidation in men.

#### 5.3.2. Resveratrol Potentially Improves Glucose Homeostasis through the Gut Microbiota

Recent studies have focused on whether the beneficial effect of resveratrol supplementation on glucose homeostasis may be mediated, at least in part, by alterations in the gut microbiota. Two recent studies carried out by the same group using a fecal microbiota transplant (FMT) show that obese mice receiving a resveratrol (0.4%)-fed mice-FMT present less Proteobacteria [201,202]; considering the association of Proteobacteria with inflammation, the authors postulated that the reduction observed could be indicative of the benefits seen in treated animals. Furthermore, they also reported decreased inflammation in the colon of FMT-recipients and suggested that bacterial metabolites or by-products of the polyphenol may be responsible for these benefits. In contrast, the authors reported decreased levels of *Akkermansia muciniphila*, a species that has been associated to improved body weight and glucose management. An interesting observation however was that resveratrol-FMT is actually more efficient than oral supplementation *per se* for the regulation of glucose homeostasis once obesity and insulin resistance are already present [201,202]. Decreased intestinal inflammation linked to resveratrol supplementation and improved glucose homeostasis has also been reported [208], in which C57Bl/6J and glucagon-like peptide-1 (GLP-1) receptor knock-out (Glp1r^-/-^) mice on a high-fat diet supplemented with 60 mg/kg/day of resveratrol for five weeks showed an increase in glucose-induced glucagon-like peptide-1 (GLP-1) and insulin secretion. This was accompanied by changes in gut microbiota composition, which suggest to be linked to the decreased intestinal inflammation in these animals. It is hypothesised that animals on a high-fat diet have increased inflammation, which leads to decreased glucose-induced insulin secretion and ultimately insulin resistance [185]. Thus, it seems that resveratrol may potentially mitigate intestinal inflammation caused by high-fat feeding via gut microbiota modulation, which could in turn increase incretin actions such as insulin and GLP-1 secretion and ameliorate glycemic control. Hence, based on the in vivo demonstration of the influence of resveratrol on the enteroendocrine axis in mice [208], a novel therapeutic action of this polyphenol should be considered.

In another study, C57Bl/6N mice with induced heart failure were administered a high dose of resveratrol (450 mg/kg/day) together with a high-fat diet for two weeks and reported a decreased Bacteroidetes-to-Firmicutes ratio in the gut microbiota and an increase in the genus Akkermansia [204]. This latter one, as previously mentioned, has been linked to improved glucose homeostasis in insulin-resistant and obese animal models, and is in contrast with what was observed in the above-discussed studies. This was accompanied by a higher abundance of the genera Parabacteroides and Bilophila, and a decrease in the Lachnospiraceae family, changes which are associated to an increased metagenomics capacity for carbohydrate metabolism, indicating a potential mechanism by which resveratrol improves insulin signaling and glucose homeostasis.

Rats under a high-fat, high-sucrose diet were supplemented with either *trans*-resveratrol (15mg/kg body weight/day) or with a combination of *trans*-resveratrol and quercetin (30mg/kg/day) for six weeks [211]. Besides a reduced weight gain, animals receiving the polyphenols exhibited lower serum insulin levels and improved insulin sensitivity. Treatments did not alter bacteria at the phylum level, however *trans*-resveratrol did reduce significantly the *Graciibacteraceae* family, the *Parabacteroides* genus, and the species *Clostridium aldenense*, *Clostridium hathewayi*, *Clostridium sp. C9, Clostridium sp. MLG661*, *Gracilibacter thermotolerans* and *Parabacteroides distasonis*, as well as increasing significantly the relative abundance of *Clostridium sp. XB90* versus animals on a high-fat, high-sucrose diet alone. Even though these changes were accompanied by a decrease in HOMA-IR, the authors could not conclude a direct association between the changes observed in gut microbiota and the beneficial effects seen in insulin resistance.

#### 5.3.3. Effects on Cardiovascular Health

Cardiovascular health is closely related to metabolic syndrome, which, as described above, is characterized by abdominal fat, high glucose, and triglyceride levels, low high-density lipoprotein cholesterol levels, and hypertension [214]. Thus, determining therapies to promote a healthy cardiovascular system will inevitably lead to an improvement of the metabolic syndrome outcome. Within this context, one group showed a protective effect of resveratrol on atherosclerosis by supplementing C57BL/6J and ApoE^-/-^ mice with 0.4% resveratrol. Supplementation increased levels of the genera Lactobacillus and Bifidobacterium, and decreased gut microbial trimethylamine production through changes in the gut microbiota, hence leading to inhibited trimethylamine-n-oxide synthesis and reduced atherosclerosis [203]. Interestingly, another study investigated the effect of supplementing Sprague Dawley rats with 50 mg/L of resveratrol in their drinking water during pregnancy and lactation whilst on a high-fructose diet [212]. They observed that the offspring from animals receiving resveratrol presented a restored systolic and diastolic blood pressure versus animals not receiving supplementation, which was elevated compared to control rats. This was accompanied by changes in gut microbiota since animals receiving a high-fructose diet presented a decreased Firmicutes-to-Proteobacteria ratio, which was normalized by resveratrol intake. Furthermore, it seems that supplementation increased the relative abundance of *Lactobacillus* and *Bifidobacterium* species, which was in accordance with that observed by Chen et al. [203], thus counteracting the impact of the high-fructose diet [212]. However, further studies are needed to determine whether such changes can be directly attributed to the beneficial impact of resveratrol on hypertension.

#### 5.3.4. Resveratrol Analogues and Gut Microbiota

Due to the reported low bioavailability of resveratrol, a few studies have been carried out to explore the potential effects of analogs such as piceatannol [215], a polyphenol that presents better bioavailability. However, the studies published so far present differing results. On the one hand, one group showed that the administration of piceatannol (15 or 45 mg/kg/day for six weeks) to Zucker (*fa/fa*) rats has little effect on gut microbiota, as well as no major impact on body weight, body fat, and glucose and lipid metabolic parameters, other than a decrease in circulating non-esterified fatty acids and in fecal *Clostridium hathewayi*, belonging to the butyrate-produce cluster Clostridium XIVa [210]. On the other hand, another study showed that 0.25% piceatannol supplementation under a high-fat diet reduced the body weight of C57Bl/6N mice, and that both 0.1% resveratrol and 0.1% and 0.25% piceatannol were able to partially prevent the increase of body fat content and adipocyte size compared to a high-fat diet alone [206]. This was accompanied by significant changes in gut microbiota, where animals supplemented with piceatannol particularly recovered from the alterations caused by a high-fat diet, which intriguingly consisted of a decreased Firmicutes abundance and increased Bacteroidetes [206]. The differences in the results between these two studies could be due to a variety of reasons, such as different concentrations and length of supplementation, together with different metagenomic workflow. Furthermore, the results of the latter study, reporting a piceatannol-induced increase in Firmicutes versus animals on a high-fat diet deserves further confirmation, since it has been well documented that this dietary intervention, revealing an obese phenotype, is generally associated to dysbiosis and lower bacterial diversity [197]. Otherwise, the rapid action of piceatannol, which results in lowering blood glucose 1 h after acute oral administration in *db*/*db* mice (50 mg/kg body weight), indicates that not all the beneficial effects of stilbenoids are mediated by a modulatory action effect on gut microbiota [144]. Together with the interactions between stilbenoids and bacteria, changes at the intestinal level and even in other targets, warrant further study regarding the potential of polyphenols for the chronic treatment of metabolic syndrome. However, such future investigations should not consider these metabolites as inactive; as mentioned above and as illustrated by the case of urolithin A, a major ellagitannin metabolite, they are endowed with noticeable health benefits [216].

## 6. Future Directions

When reflecting on the impact a certain compound has on health, in this case, resveratrol, and whether its effects may be mediated by gut microbiota, many questions appear: Is gut microbiota at the base of energy regulation? Could resveratrol be having a significant impact on it, making it relevant to consider it a prebiotic in the future? Thus, there are still many issues to consider and investigate in the near future.

As recently reviewed [217], the modulation of gut microbiota is being considered as a key method to treat obesity. However, it is seen that the efficiency of this strategy is inconsistent due to inherent differences in gut microbiota among individuals. Furthermore, it is yet to be determined what an “ideal” gut microbiota composition is and how to effectively manipulate it.

Although the results so far are promising, further studies are warranted in both animals and humans. It seems that resveratrol does have an effect on gut microbiota that was initially unsuspected when, more than a decade ago, the stilbenoid was found to modulate energy balance. Now, resveratrol is considered as a potential prebiotic candidate to promote changes in bacterial composition associated with a healthy phenotype. However, whether this interaction is involved in several of the beneficial health effects attributed to this polyphenol is yet to be elucidated. At present, and to the best of our knowledge, most studies are still observational, and nearly all in animal models, presenting interesting correlations but few mechanistic clues as to how the resveratrol-gut microbiota-metabolism axis could be functioning. As discussed above, it seems that in the past 10 years most studies have shown associations between gut microbiota and health and disease, but they have failed to prove a direct causal relationship [154]. One of the potential mechanisms put forward by Nohr et al. [218] could be that resveratrol reverses or inhibits the effects of Gram-negative bacteria-derived LPS in the gut, thereby preventing an alteration of the intestinal epithelium permeability, and consequently decreasing endotoxemia of intestinal origin, low-grade inflammation, and obesity. However, more studies are needed in order to confirm this interesting hypothesis. Another future route of study could be to analyze the potential effects of resveratrol on SCFA in the gut since recent studies seem to be pointing to the by-products of resveratrol as the key actors, including SCFA, already known to play a regulatory role in energy homeostasis.

At this stage, and considering the observations made in different models, we hypothesize two putative mechanisms of action of resveratrol on the interactions between bacteria and intestinal mucosa.

First, resveratrol may influence the turnover of SCFA and various intraluminal lipids by modulating both bacterial production and handling in the intestine. Indeed, resveratrol has been reported to exert an important anti-lipogenic effect on an in vitro model of the human small intestinal mucosa, in which epithelial cells were treated with LPS with or without prior challenge with resveratrol [219]. Among the genes found to be regulated by LPS but repressed by resveratrol were endothelial lipase, acyl-CoA synthetase, and many others involved in lipid synthesis and/or cholesterol handling. Furthermore, resveratrol has been reported to inhibit lipogenesis in rodent fat cells too, in an acute, short-term manner. Taking together these observations, it could be suggested that resveratrol can directly reduce the activity of enzymes involved in lipogenic pathways, in a concerted manner with the down-regulatory role it plays on their expression [143]. All these integrated actions of resveratrol limit LPS bacterial production and its consequences on epithelial transcript factors, reshape intestinal lipid metabolism, and lead to a reinforcement of the intestinal barrier when challenged by an excess of lipids. As a consequence of such modulation, the beneficial effects of ingested resveratrol or its metabolites before and after trans-epithelial absorption seemingly include changes in the intraluminal microbiota and SCFA levels, gut barrier integrity, and blood cholesterol and triglycerides.

Our second hypothesis is that the antioxidant properties of polyphenols can trigger pleiotropic responses in both the microbiota and the host. It is worthy to note that NADPH oxidase was upregulated by LPS but down-regulated by resveratrol in epithelial cells used in the abovementioned study [219]. NADPH oxidase is a complex membrane-bound enzyme implicated in the immune response, but it is also recognized as generating superoxides and ROS. Because of their antioxidant properties, resveratrol and its derivatives can potentially counteract the consequences of ROS produced by NADPH oxidase and other ROS-generating enzymes. In this sense, we recently confirmed that resveratrol impairs not only the fate of hydrogen peroxide generated by monoamide oxidase in fat cells but also the catalytic activity of the oxidase itself [143]. In bacteria, there are amine oxidases equivalent to those found in mammalian cells. Although their roles are not completely defined, it has been proposed that they are useful for survival in harsh culture conditions, allowing for diversification of the sources of nitrogen and provide a growth advantage over competing species (via the hydrogen peroxide they produce). Noteworthy, the deletion of the *Escherichia coli* copper amine oxidase (ECAO) has been demonstrated to alter the growth abilities of its associated strain, with significant metabolic changes [220]. Many other alterations have been reported among strains expressing (or not) ECAO and have led to this enzyme being characterized as capable of influencing bacterial growth and adhesion [220]. Thus, in addition to its well-known antimicrobial properties, resveratrol might reshape gut bacteria composition by counteracting ROS actions and inhibiting amine oxidase activities, thereby selecting given bacterial species. However, whether this potential mechanism is different from other dietary antioxidants and contributes to the specificity of the stilbenoid in reversing gut microbial dysbiosis remains to be elucidated.

As a final note, it is important to mention that differences in results among the studies discussed may be due to a variety of reasons: Species (mice vs. rats vs. humans) and disease model (knock-out, obese, etc.); a wide variety in dosage of resveratrol and duration of supplementation; the administration route (mixed in diet or drinking water, oral gavage); and the combination with other compounds (e.g. quercetin), making it difficult to dissect the effects of each. Furthermore, studies also differ in how they report the changes observed in gut microbiota composition, where differences are seen in either species, genus, and/or the phylum level, making it tricky to elucidate the impact of polyphenol-microbiota interactions in the first instance.

## 7. Conclusions

It is becoming clear that we are moving towards an era in which treatments and strategies, particularly nutritional interventions such as resveratrol supplementation, to counteract obesity and metabolic syndrome will need a personalized approach tailored to the individual in order to be as effective as possible. The gut microbiota composition is an important factor to consider in this equation. Further studies, together with growing knowledge on the role of gut microbiota composition, will inevitably provide exciting future directions, as in the case of resveratrol-based supplementations.

## Figures and Tables

**Table 1:** Review of studies analyzing the effect of resveratrol on gut microbiota composition.

Species	Resveratrol Dose	Duration	Modulation of Gut Microbiota	Effects on Metabolic Syndrome Alterations	
C57Bl/6N mice	0.4% resveratrol (+FMT)	2–8 weeks	↑ *Bacteroides* and *Parabacteroides* ↓ *Turicibacteraceae, Moryella, Lachnospiraceae* and *Akkermansia*	FMT from resveratrol-fed mice improved glucose homeostasis and lowered blood pressure.	[201,202]
C57Bl/6J and ApoE^−/−^ mice	0.4% resveratrol	1 or 2 months	↑ *Lactobacillus* and *Bifidobacterium*	Inhibition of TMAO synthesis and reduced atherosclerosis.	[203]
C57Bl/6N mice	450 mg/kg/day	2 weeks	↓ Bacteriodetes-to-Firmicutes ratio ↑ Parabacteroides, Bilophila and Akkermansia ↓ Lachnospiraceae	Increased skeletal muscle insulin sensitivity, glucose utilization and metabolic rate.	[204]
C57Bl/6J mice	200 mg/kg/day	8 weeks	↓ *Lactococcus*, *Clostridium XI*, *Oscillibacter* & *Hydrogenoanaerobacterium*	Reduced fat deposition and body weight gain.	[205]
C57Bl/6 mice	0.1% resveratrol, 0.1% piceatannol or 0.25% piceatannol	18 weeks	Piceatannol: ↑ Firmicutes, Clostridiales, Shpingobacteriales, Blautia, *P. kwangyangensis* & *Lactobacillus* ↓ Bacteroidetes	Piceatannol: Reduced body weight, perigonadal adipose tissue, adipocyte size, plasma glucose and cholesterol Resveratrol: Reduced perigonadal adipose tissue, adipocyte size	[206]
Kunming mice	200 mg/kg/day	12 weeks	↑ Bacteroidetes, *Lactobacillus* & *Bifidobacterium* ↓ Firmicutes and *Enterococcus faecalis*	Decreased body and visceral adipose weight. Lower plasma glucose and lipid levels.	[207]
C57Bl/6J and Glp1r^-^ mice	60 mg/kg/day	5 weeks	Restored bacterial composition of animals fed a high-fat diet. ↓ *Parabacteroides jonsonii DMS 18315* (a), *Alistipes putredinis DMS 17216* (b) and *Bacteroides vulgatus ATCC 8482*	Reduced glucose intolerance in diabetic mice without affecting fasting glycemia.	[208]
Wistar rats	Quercetin (30 mg/kg/day) and resveratrol (15 mg/kg/day)	10 weeks	↑ *Bacteroidales S24-7 group*, *Christensenellaceae*, *Akkermansia muciniphila*, *Ruminococcaceae, Ruminococcaceae UCG-014* & *Ruminococcaceae UCG-005* ↓Firmicutes & Firmicutes-to-Bacteroidetes ratio	Lower body weight gain and adipose tissue weight.	[209]
Zucker rat (*fa/fa*)	Piceatannol (15 and 45 mg/kg/day)	6 weeks	↓ *Clostridium hathewayi*	No impact on body weight and body fat, glucose, and lipid metabolic parameters.	[210]
Wistar rats	*Trans*-resveratrol (15 mg/kg/day) or *trans*-resveratrol + quercetin (30 mg/kg/day)	6 weeks	*Trans*-resveratrol & quercetin: ↓Firmicutes-to-Bacteroidetes ratio ↓*Erysipelotrichaceae, Bacillus, Eubacterium cylindroides*	Improved HOMA-IR and insulin sensitivity.	[211]
Sprague Dawley rats	50 mg/L of resveratrol 50 mg/L	3 months	↑ Firmicutes-to-Proteobacteria ratio	Restored systolic and diastolic blood pressure.	[212]
Humans	EGCG (282 mg/day) + resveratrol (80 mg/day)	12 weeks	Men: ↓ Bacteroidetes	Increased fat oxidation and skeletal muscle mitochondrial oxidative capacity.	[213]

FMT, fecal microbiota transplant; TMAO, trimethylamine N-oxide; HOMA-IR, homeostatic model assessment–insulin resistance; HF, high-fat; ND, normal-diet; EGCC, epigallocatechin-3-gallate; ↑, increase; ↓, decrease.
